# Gray Matter Volume Variability in Young Healthy Adults: Influence of Gender Difference and Brain-Derived Neurotrophic Factor Genotype

**DOI:** 10.1093/cercor/bhab370

**Published:** 2021-10-11

**Authors:** Hiraku Watanabe, Sho Kojima, Kazuaki Nagasaka, Ken Ohno, Noriko Sakurai, Naoki Kodama, Naofumi Otsuru, Hideaki Onishi

**Affiliations:** Graduate School, Niigata University of Health and Welfare, Niigata-City, Niigata, 950-3198, Japan; Institute for Human Movement and Medical Sciences, Niigata University of Health and Welfare, Niigata-City, Niigata, Niigata, 950-3198, Japan; Division of Physical Therapy and Rehabilitation Medicine, University of Fukui Hospital, Yoshida-gun, Fukui, 910-1193, Japan; Institute for Human Movement and Medical Sciences, Niigata University of Health and Welfare, Niigata-City, Niigata, Niigata, 950-3198, Japan; Department of Physical Therapy, Niigata University of Health and Welfare, Niigata City, Niigata, 950-3198, Japan; Institute for Human Movement and Medical Sciences, Niigata University of Health and Welfare, Niigata-City, Niigata, Niigata, 950-3198, Japan; Department of Physical Therapy, Niigata University of Health and Welfare, Niigata City, Niigata, 950-3198, Japan; Institute for Human Movement and Medical Sciences, Niigata University of Health and Welfare, Niigata-City, Niigata, Niigata, 950-3198, Japan; Department of Radiological Technology, Niigata University of Health and Welfare, Niigata City, Niigata, 950-3198, Japan; Institute for Human Movement and Medical Sciences, Niigata University of Health and Welfare, Niigata-City, Niigata, Niigata, 950-3198, Japan; Department of Radiological Technology, Niigata University of Health and Welfare, Niigata City, Niigata, 950-3198, Japan; Institute for Human Movement and Medical Sciences, Niigata University of Health and Welfare, Niigata-City, Niigata, Niigata, 950-3198, Japan; Department of Radiological Technology, Niigata University of Health and Welfare, Niigata City, Niigata, 950-3198, Japan; Institute for Human Movement and Medical Sciences, Niigata University of Health and Welfare, Niigata-City, Niigata, Niigata, 950-3198, Japan; Department of Physical Therapy, Niigata University of Health and Welfare, Niigata City, Niigata, 950-3198, Japan; Institute for Human Movement and Medical Sciences, Niigata University of Health and Welfare, Niigata-City, Niigata, Niigata, 950-3198, Japan; Department of Physical Therapy, Niigata University of Health and Welfare, Niigata City, Niigata, 950-3198, Japan

**Keywords:** brain-derived neurotrophic factor genotype, gender, gray matter volume, voxel-based morphometry

## Abstract

Although brain gray matter (GM) plastically changes during short-term training, it is still unclear whether brain structures are stable for short periods (several months). Therefore, this study aimed to re-test the short-term variability of GM volumes and to clarify the effect of factors (gender and BDNF-genotype) expected to contribute to such variability. The subjects comprised 41 young healthy adults. T1-weighted images were acquired twice with an interval of approximately 4 months using a 3 T-MRI scanner. Voxel-based morphometry (VBM) was used to calculate GM volumes in 47 regions. The intraclass correlation coefficient (ICC) and Test–retest variability (%TRV) were used as indices of variability. As a result, the ICCs in 43 regions were excellent (ICC > 0.90) and those in 3 regions were good (ICC > 0.80), whereas the ICC in the thalamus was moderate (ICC = 0.694). Women had a higher %TRV than men in 5 regions, and %TRV of the Val66Val group was higher than that of the Met carrier group in 2 regions. Moreover, the Female-Val66Val group had a higher %TRV than the Male-Met carrier group in 3 regions. These results indicate that although the short-term variability of GM volumes is small, it is affected by within-subject factors.

## Introduction

Recently, the development of brain structure analysis methods has made possible the visualization of plastic changes in the human brain gray matter (GM) volumes. Voxel-based morphometry (VBM) is one of the commonly used methods for structural brain imaging analysis. This approach enables the calculation of the gray and white matter volumes, just as well as that of the cerebrospinal fluid using T1-weighted imaging recorded using magnetic resonance imaging (MRI) (Ashburner and Friston 2000). A longitudinal study using VBM was applied to assess plastic changes in the gray matter volumes. For example, juggling training for 4 weeks increases the GM volumes in the area of V5/hMT (Driemeyer et al. 2008). Furthermore, the cognitive learning for 2 weeks increases the GM volumes in the dorsomedial frontal cortex, orbitofrontal cortex, and precuneus (Ceccarelli et al. 2009). Therefore, VBM measurements reportedly enable the detection of GM volume plastic changes after intensive training for a short-term period.

A review focusing on VBM recommends using an experimental design with an age-matched control group when applying VBM in longitudinal studies as changes in the GM volumes should be considered over time (Lovden et al. 2013). However, multiple studies analyze only the time factor of the intervention group, examined without a control group (Driemeyer et al. 2008; Teutsch et al. 2008; Granert et al. 2011; Landi et al. 2011; Hamzei et al. 2012; Stein et al. 2012). Changes in the GM volumes over a short-term period are assumably small if no comparison is made between the intervention and control groups, as in these previous studies. However, GM volume variability during a short-term period remains elusive.

Gender is a factor that affects the variability of GM volumes. Women exhibit variable neuronal activities such as the balance of excitatory and inhibitory neural activity (Smith et al. 1999; Hattemer et al. 2007; Schloemer et al. 2020) and a neural network (Petersen et al. 2014; Lisofsky et al. 2015) with changes in the menstrual cycle. Therefore, women presumably display high GM volume variability in short-term periods. Moreover, brain-derived neurotrophic factor (BDNF) genotype is also a factor that affects GM volume variability. BDNF is a protein important for the growth and divergence of neurons (Poo 2001; Park and Poo 2013) and the development of hippocampal neurons inhibited in BDNF knockout mice (Gao et al. 2009). There is a mutant form of the gene that controls BDNF activity in which valine (Val) is mutated to methionine (Met) at codon 66 (a patient with such a mutation is referred to as a “Met carrier”). Single nucleotide polymorphisms (Val66Met) and Met homozygotes (Met66Met) exhibit reportedly reduced BDNF activity and neuronal plasticity compared to wild type (Val66Val) (Egan et al. 2003; Kleim et al. 2006; McHughen et al. 2010; Cirillo et al. 2012). Therefore, Val66Val presumably exhibits high GM volume variability due to high neural plasticity.

Seiger et al. reported that the GM volume variability of healthy young adults for 3 months was small. However, the sample size comprised only 10 subjects (Seiger et al. 2015). Furthermore, the factors influencing the individual differences in GM volume variability have not been investigated. Therefore, it is necessary to increase the sample size and examine the effects of within-subject factors that might contribute to individual differences in variability in order to examine the variability of GM volumes in detail over a short-term period. Therefore, this study aimed to re-test the short-term variability of GM volumes in young healthy adults and to clarify the effects of factors (Gender and BDNF-genotype) expected to contribute to the GM volume variability.

**Table 1:** Subjects information

	Total subjects	Female-Val66Val	Female-Met carrier	Male-Val66Val	Male-Met carrier
The number of subjects	41	9	12	8	12
Age (mean ± SD)	22.1 ± 2.2	22.1 ± 2.2	21.3 ± 0.7	22.8 ± 2.7	23.0 ± 3.3
Handedness (Lt/Rt)	3/38	1/8	0/12	2/6	0/12
Days between Test 1 and Test 2 (Mean ± SD)	114.5 ± 42.8	111.9 ± 40.4	101.6 ± 25.7	124.6 ± 58.8	132.5 ± 50.1

## Material and Methods

### Participants

Overall, 41 healthy volunteers (aged 20–23 years, mean ± standard division (SD): 22.1 ± 2.2 years; 20 men and 21 women) participated in this study (Table 1). None of the participants reported taking any drugs or medications, which could affect the central nervous system function. This study was approved by the Ethics Committee of Niigata University of Health and Welfare and was conducted in accordance with the Declaration of Helsinki. All participants provided written informed consent before participation.

### Experimental Procedure

The T1-weighted images were acquired twice (first: Test 1; second: Test 2) with an interval of approximately 4 months (mean ± SD: 114.5 ± 42.8 days; longest interval: 222 days; shortest interval: 44 days). All experiments were performed in the morning.

### T1-Weighted Image Acquisition

The T1-weighted images were acquired using a 3 T Vantage Galan MRI scanner (Canon Medical Systems, Tochigi, Japan) with a 32-channel head coil (QD coil, 32ch head SPEEDER, Atlas SPEEDER head/neck). The head was fixed with a sponge to prevent motion artifacts. Moreover, headphones were used to reduce the influence of the imaging noise of the MRI scanner. Magnetization-prepared rapid gradient-echo (MPRAGE) was used as the imaging sequence (repetition time: 5.8 ms, echo time: 2.7 ms, inversion time: 900 ms, field of view: 23 × 23, slice: 160 slices, slice thickness: 1.2 mm, scan matrix: 256 mm × 256 mm, slice gap: non-gap, flip angle: 9). The structural brain imaging analysis was performed using T1-weighted images constructed from the horizontal plane.

### BDNF Genotyping

The BDNF genotyping was performed based on the SNP database (BDNF-rs6265) of the National Center for Biotechnology Information. The polymerase chain reaction was used for BDNF genotyping as described previously (Onishi et al. 2018; Sasaki et al. 2021).

**Figure 1:**
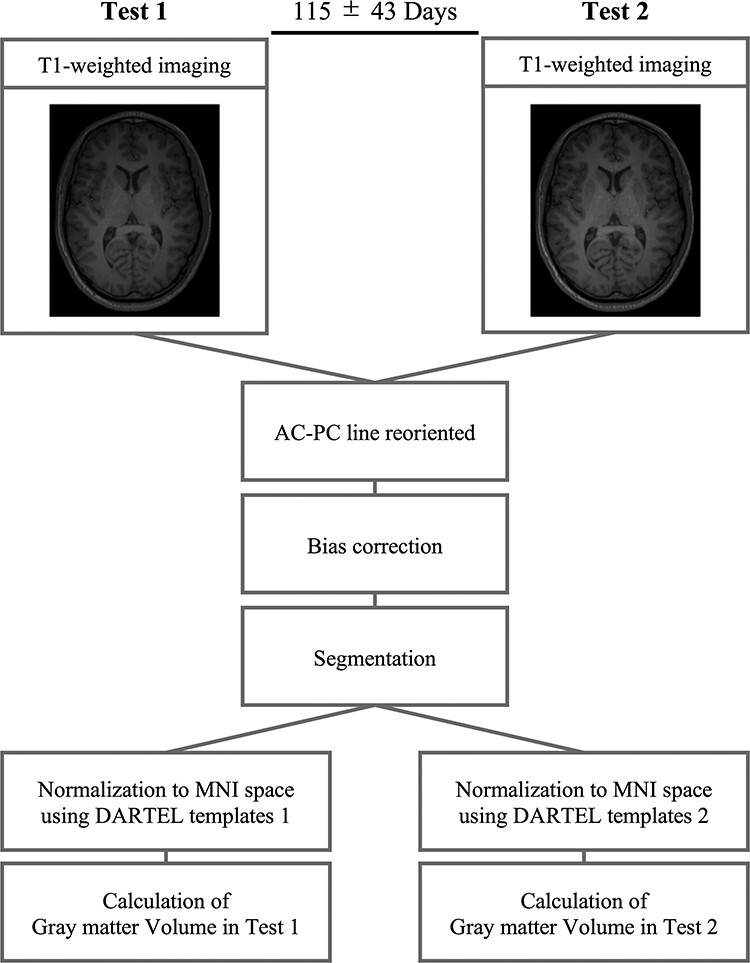
Voxel-based morphometry analysis. T1-weighted images were acquired twice (first: Test 1; second: Test 2) with an interval of approximately 4 months (mean ± SD: 114.5 ± 42.8 days). The T1-weighted images used for analysis were visually checked for motion artifacts. The AC-PC line was collected automatically. The T1-weighted images were segmented into GM, white matter, and cerebrospinal fluid after bias correction. The spatial normalization to the MNI space was performed using the DARTEL algorithm. The template image used for DARTEL was created using 41 images measured in each test (Test1: Template 1, Test 2: Template 2). The GM volumes were calculated from the preprocessed T1-weighted brain images of Tests 1 and 2.

### Voxel-Based Morphometry Analysis

The GM volumes were calculated using the VBM methods. VBM was performed using Matlab R2020a (MathWorks, Natick, MA) and the VBM-toolbox driven by SPM12 (http://www.fil.ion.ucl.ac.uk/spm). The T1-weighted images used for the analysis were visually checked for motion artifacts. The AC-PC line was collected automatically using the “auto_reorient” programming. The T1-weighted images were segmented into GM, white matter, and cerebrospinal fluid after bias correction (Bias regularization: very light; Bias FWHM cutoff: 60 mm). Spatial normalization was performed using the Diffeomorphic Anatomical Registration Through Exponentiated Lie Algebra (DARTEL) algorithm. The template image used for DARTEL was created based on 41 images in Tests 1 and 2 (Templates 1 and 2), respectively. The total GM, white matter, and cerebrospinal fluid volumes were calculated from the pre-processed T1-weighted images. The sum of the total GM and white matter volumes were calculated as the total brain volume. The GM volumes were calculated using the “get_totals” programming. The regional GM volume of each brain region was calculated using the mask image created using the “wfu_pickatlas” programming with the “get_totals” programming. All 47 mask images were created based on the Automatic Anatomical Labeling (AAL) (Tzourio-Mazoyer et al. 2002). We combined the left and right brain regions as we assumed that the GM volume variability between the cerebral hemispheres would be small (Seiger et al. 2015) (Fig. 1).

### Statistical Analysis

Statistical analyses were performed using the SPSS statistics 25 software (IBM SPSS, Armonk, New York, USA). The intraclass correlation coefficient (ICC) was used as a measure of the GM volume variability between Tests 1 and 2. ICC is an index calculated between 0 and 1. The degree of variability was classified in 3 categories: moderate (0.50–0.75), good (0.75–0.90), and excellent (0.90–1.00) (Koo and Li 2016). Test–retest variability (%TRV) was used as an index to compare individual differences in GM volume variability (Seiger et al. 2015; Jing et al. 2018; Yan et al. 2020). The %TRV was defined as the absolute value of the difference between the GM volumes of Tests 1 and 2 divided by the average of the GM volumes of Tests 1 and 2. In other words, a high %TRV indicates high variability. The %TRV of each brain region was compared between two groups (women vs. men or Val66Val vs. Met carrier) using the Mann–Whitney test. In addition, the %TRV of each brain region was compared between four groups (Female-Val66Val vs. Female-Met carrier vs. Male-Val66Val vs. Male-Met carrier) using the Kruskal–Wallis test. Post hoc analyses were performed using the Dunn–Bonferroni test if the null hypothesis was rejected. The statistical significance was set at *P* < 0.05. Post hoc power analyses were performed using G*Power (version 3.1.9.6). Power (1 − β) was considered high when it was >0.80 (Cohen 1992).

## Results

**Figure 2:**
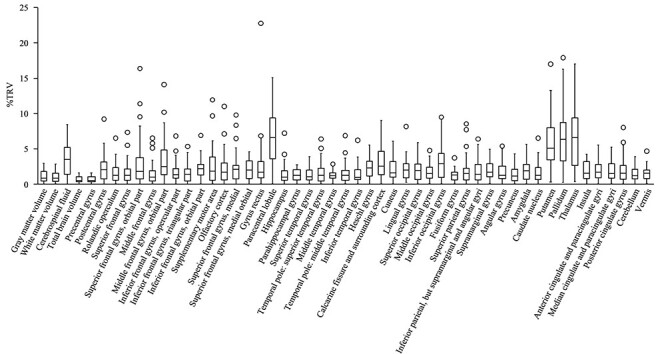
%TRV. Box-and-whisker plots created using the %TRV of all subjects. The median %TRV for each brain region was less than 3% in 43 of 47 regions, while the %TRV of the paracentral lobule, putamen, pallidum, and thalamus was more than 3%.

Table 2 shows the median %TRV (first quartile, third quartile) and ICC of the GM, white matter, cerebrospinal fluid, and total brain volumes. Figure 2 shows a box-and-whisker diagram based on the %TRV of the GM volumes. The median %TRV of each brain region was 0.98–6.61%. The mean %TRV of the 47 regions was 2.01%. The median %TRV in 43 of 47 regions (91%) was less than 3%, while the %TRV of the paracentral lobule, putamen, pallidum, and thalamus was more than 3%. Moreover, testing the relationship between %TRV and imaging interval using Spearman’s rank correlation coefficient revealed that there was no significant relationship (*r* = −0.199, *P* = 0.211).

**Table 2:** %TRV of total GM volume, total white matter volume, spinal cord volume, total brain volume, and regional GM volume

	ICC	Total subjects Median (Q1, Q3)	Female-Val66Val Median (Q1, Q3)	Female-Met carrier Median (Q1, Q3)	Male-Val66Val Median (Q1, Q3)	Male-Met carrier Median (Q1, Q3)	Kruskal-wallis test *P*-value
**Total**							
Gray matter volume	0.993	0.87 (0.45, 1.82)	1.54 (0.72, 2.38)	1.18 (0.74, 1.58)	0.98 (0.47, 1.98)	0.45 (0.30, 1.01)	0.154
White matter volume	0.995	0.84 (0.44, 1.56)	0.58 (0.44, 1.60)	1.29 (0.80, 1.72)	0.75 (0.37, 1.16)	0.56 (0.26, 1.22)	0.236
Cerebrosspinal fluid	0.979	3.50 (1.39, 5.22)	6.18 (3.69, 6.94) *	2.38 (1.32, 4.34)	4.65 (3.21, 5.61)	1.50 (0.21, 2.96)	0.010
Total brain volume	0.996	0.49 (0.35, 1.07)	1.07 (0.45, 1.30) *	0.48 (0.05, 0.86)	0.62 (0.49, 0.81)	0.39 (0.18, 0.46)	0.047
**Central region**							
Precentral gyrus	0.953	2.07 (0.74, 3.18)	2.22 (0.45, 3.81)	2.57 (0.80, 3.49)	1.91 (1.46, 2.51)	1.80 (0.53, 2.71)	0.773
Postcentral gyrus	0.979	1.28 (0.57, 2.34)	2.00 (0.19, 3.20)	1.55 (1.02, 2.73)	1.72 (1.05, 2.50)	0.67 (0.50, 1.29)	0.405
Rolandic operculum	0.970	1.27 (0.54, 2.16)	2.34 (0.92, 4.04)	1.29 (0.94, 1.71)	1.45 (0.56, 2.39)	0.65 (0.54, 1.35)	0.297
**Frontal lobe**							
Superior frontal gyrus	0.959	1.51 (0.75, 3.35)	1.82 (1.18, 6.84)	1.56 (0.63, 2.74)	1.21 (0.76, 2.85)	1.84 (1.07, 3.51)	0.775
Superior frontal gyrus, orbital part	0.916	2.24 (0.76, 3.85)	4.29 (0.76, 4.66)	1.46 (0.48, 2.71)	2.70 (1.45, 3.84)	2.10 (1.55, 4.38)	0.554
Middle frontal gyrus	0.976	1.03 (0.46, 1.92)	1.39 (0.46, 6.01)	1.27 (0.56, 2.48)	0.84 (0.78, 1.20)	1.01 (0.37, 1.17)	0.459
Middle frontal gyrus, orbital part	0.936	2.53 (1.36, 4.84)	4.28 (1.57, 8.68)	2.02 (0.71, 3.63)	2.00 (0.14, 4.86)	2.64 (2.42, 3.98)	0.314
Inferior frontal gyrus, opercular part	0.967	1.37 (0.85, 2.19)	1.94 (1.08, 3.74) *	2.18 (1.30, 3.39) *	1.27 (0.59, 1.58)	0.81 (0.55, 1.05)	0.005
Inferior frontal gyrus, triangular part	0.979	1.43 (0.48, 2.14)	2.08 (1.68, 2.42)	1.39 (0.73, 1.84)	1.87 (0.52, 2.28)	0.79 (0.26, 1.17)	0.172
Inferior frontal gyrus, orbital part	0.975	2.20 (1.32, 2.79)	2.77 (1.32, 2.89)	1.35 (0.91, 2.29)	2.43 (2.26, 3.03)	2.13 (1.41, 2.30)	0.135
Supplementary motor area	0.943	1.84 (0.55, 3.84)	2.28 (0.44, 6.08)	1.99 (0.82, 3.63)	1.82 (1.27, 3.08)	2.10 (0.47, 4.51)	0.988
Olfactory cortex	0.956	1.73 (0.62, 2.99)	1.94 (1.19, 2.99)	1.62 (0.75, 2.20)	1.71 (0.44, 3.09)	1.69 (0.77, 2.92)	0.905
Superior frontal gyrus, medial	0.962	2.11 (0.74, 2.64)	2.28 (0.12, 4.04)	2.15 (1.34, 3.42)	1.58 (0.58, 2.35)	1.14 (0.77, 2.58)	0.764
Superior frontal gyrus, medial orbital	0.980	2.03 (0.84, 3.29)	2.22 (1.75, 4.21)	0.87 (0.29, 2.12)	3.31 (1.03, 3.71)	2.32 (1.37, 2.72)	0.104
Gyrus rectus	0.917	1.71 (0.93, 3.21)	1.64 (0.41, 5.40)	1.42 (0.89, 2.00)	1.76 (0.95, 2.35)	2.78 (1.03, 4.07)	0.765
Paracentral lobule	0.813	6.61(3.61, 9.38)	4.71 (3.71, 8.10)	7.43 (4.25, 9.61)	3.35 (2.38, 6.89)	8.20 (4.47, 10.47)	0.232
**Temporal lobe**							
Hippocampus	0.971	1.02 (0.54, 1.92)	1.86 (0.76, 3.49)	1.46 (0.80, 2.00)	0.83 (0.61, 1.12)	0.88 (0.28, 1.59)	0.371
Parahippocampal gyrus	0.982	1.24 (0.62, 2.05)	1.90 (0.21, 2.54)	1.29 (0.65, 1.94)	1.16 (0.83, 1.50)	1.36 (0.95, 1.92)	0.776
Superior temporal gyrus	0.983	1.04 (0.60, 1.99)	2.56 (1.96, 2.93) *	1.23 (0.57, 1.72)	0.73 (0.39, 1.65)	0.86 (0.55, 1.06)	0.039
Temporal pole: superior temporal gyrus	0.970	1.27 (0.44, 2.24)	1.34 (0.88, 2.24)	1.56 (0.45, 2.02)	0.81 (0.28, 2.56)	1.60 (0.46, 2.43)	0.855
Middle temporal gyrus	0.988	1.21 (0.86, 1.61)	1.24 (0.98, 1.45)	0.96 (0.59, 1.65)	1.61 (1.45, 2.21)	0.88 (0.46, 1.45)	0.169
Temporal pole: middle temporal gyrus	0.983	1.24 (0.58, 1.95)	1.03 (0.53, 1.36)	1.19 (0.82, 1.69)	1.72 (1.39, 3.08)	1.21 (0.56, 2.33)	0.320
Inferior temporal gyrus	0.987	0.98 (0.66, 2.04)	0.82 (0.56, 2.04)	1.01 (0.46, 1.35)	1.03 (0.69, 2.76)	1.09 (0.91, 2.27)	0.496
Heschl gyrus	0.960	2.29 (1.16, 3.23)	2.10 (0.67, 2.75)	2.04 (1.22, 3.15)	2.79 (1.74, 3.85)	2.27 (1.28, 3.16)	0.768
**Occipital lobe**							
Calcarine fissure and surrounding cortex	0.941	2.55 (1.35, 4.65)	2.54 (1.35, 4.51)	3.79 (1.12, 5.33)	3.35 (1.81, 5.23)	2.24 (1.21, 3.66)	0.565
Cuneus	0.972	1.60 (1.00, 3.22)	2.39 (1.06, 2.94)	1.74 (1.06, 2.24)	3.59 (1.23, 4.99)	1.00 (0.40, 1.93)	0.101
Lingual gyrus	0.952	1.99 0.90, 2.92)	2.29 (1.99, 3.17)	2.34 (0.65, 4.24)	2.11 (1.23, 2.31)	1.11 (0.78, 2.10)	0.351
Superior occipital gyrus	0.976	1.97 (0.65, 2.88)	1.24 (0.58, 2.73)	2.17 (0.44, 3.37)	2.62 (1.94, 3.43)	1.59 (1.09, 2.10)	0.696
Middle occipital gyrus	0.989	1.52 (0.88, 2.40)	1.86 (1.26, 2.20)	1.76 (1.45, 3.14)	0.99 (0.51, 1.38)	1.53 (0.84, 2.36)	0.103
Inferior occipital gyrus	0.970	2.90 (0.98, 4.36)	4.21 (1.17, 4.56)	3.09 (1.55, 5.22)	2.25 (0.64, 4.30)	2.70 (0.50, 3.52)	0.619
Fusiform gyrus	0.985	1.32 (0.60, 1.73)	1.23 (1.17, 1.48)	1.42 (1.02, 1.66)	1.61 (0.54, 2.12)	0.73 (0.22, 1.55)	0.436
**Parietal lobe**							
Superior parietal gyrus	0.975	1.52 (0.61, 2.25)	1.05 (0.44, 1.96)	1.27 (0.36, 1.69)	1.99 (1.20, 4.36)	1.82 (0.63, 2.28)	0.449
Inferior parietal, but supramarginal and angular gyri	0.967	1.43 (0.61, 2.79)	1.20 (0.50, 1.88)	1.43 (0.61, 3.12)	1.38 (0.25, 3.14)	1.78 (1.33, 2.67)	0.709
Supramarginal gyrus	0.969	1.70 (1.04, 2.93)	3.45 (1.09, 4.08)	1.53 (0.88, 2.58)	1.86 (1.45, 2.49)	1.37 (0.96, 2.17)	0.312
Angular gyrus	0.981	1.28 (0.80, 2.55)	1.27 (0.71, 2.14)	1.90 (0.96, 3.03)	1.67 (0.88, 2.60)	1.07 (0.71, 1.44)	0.366
Precuneus	0.982	1.15 (0.53, 2.10)	1.95 (1.06, 2.87)	1.18 (0.42, 1.87)	1.15 (0.61, 2.10)	0.84 (0.69, 1.36)	0.381
**Subcortical**							
Amygdala	0.977	1.93 (0.72, 2.79)	0.77 (0.71, 1.91)	2.22 (1.76, 3.03)	2.16 (0.92, 3.10)	2.05 (0.59, 2.74)	0.186
Caudate nucleus	0.981	1.27 (0.66, 2.37)	1.27 (0.51, 1.68)	1.39 (0.84, 2.40)	1.88 (1.35, 3.03)	0.83 (0.24, 1.82)	0.640
Putamen	0.873	5.10 (3.43, 8.02)	3.93 (3.64, 6.51)	8.15 (6.35, 9.37)	3.11 (0.89, 4.91)	4.42 (2.37, 6.45)	0.047
Pallidum	0.805	6.36 (3.31, 8.75)	5.59 (3.40, 9.37)	8.44 (6.96, 13.41)	5.29 (3.14, 6.63)	5.65 (1.76, 8.20)	0.040
Thalamus	0.694	6.61 (2.67, 9.41)	5.22 (3.04, 8.76)	8.45 (3.54, 11.13)	7.26 (2.58, 9.71)	4.64 (2.86, 8.02)	0.871
**Insula**							
Insula	0.959	1.55 (0.82, 3.18)	2.09 (0.43, 3.75)	1.32 (0.27, 1.71)	1.50 (1.16, 2.63)	2.07 (1.50, 3.00)	0.329
**Cingulate gyrus**							
Anterior cingulate and paracingulate gyri	0.978	1.71 (0.93, 2.79)	2.41 (1.97, 3.21)	1.38 (1.02, 2.54)	2.22 (1.68, 2.87)	1.18 (0.86, 1.86)	0.296
Median cingulate and paracingulate gyri	0.968	1.49 (0.85, 2.89)	2.13 (1.91, 3.53) *	1.64 (0.95, 2.78)	1.34 (0.73, 2.99)	0.77 (0.63, 1.27)	0.016
Posterior cingulate gyrus	0.980	1.57 (0.70, 2.65)	2.15 (0.46, 2.92)	1.92 (0.56, 3.36)	1.38 (1.12, 2.54)	0.94 (0.62, 2.01)	0.623
**Cerebellum**							
Cerebellum	0.981	1.23 (0.74, 2.11)	1.16 (0.24, 2.44)	1.26 (1.04, 1.55)	1.88 (1.18, 2.53)	0.77 (0.32, 1.49)	0.242
Vermis	0.971	1.57 (0.81, 2.01)	1.62 (1.11, 2.27)	1.71 (0.92, 2.02)	1.60 (1.45, 1.89)	1.36 (0.49, 1.79)	0.666
**All ROIs Average** (**Min**—**Max**)		2.01 (0.98–6.61)	2.26 (0.77–0.59)	2.22 (0.87–8.45)	2.04 (0.73–7.26)	1.81 (0.65–8.20)	

Dann-Bonferroni Test: *P < 0.05 (vs. Male Met-carrier).

### GM Volume Variability in all Subjects

The ICC in 43 of 47 regions were excellent (ICC(1, 2) > 0.90) and 3 regions were good (paracentral lobule: ICC(1, 2) = 0.813, putamen: ICC(1, 2) = 0.873, pallidum: ICC(1, 2) = 0.805), while ICC in thalamus was moderate (ICC(1, 2) = 0.694) (Table 2).

### The Effect of Gender on GM Volume Variability

**Figure 3:**
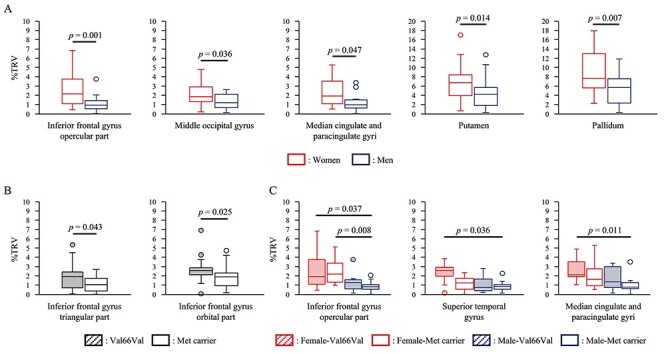
Differences in GM volume variability. (***A***) Differences in GM volume variability between women and men. Box-and-whisker diagram with red line: females, with blue line: males. The %TRV of the women was significantly higher than that of the men in 5 of 47 regions. (***B***) Differences in GM volume variability between Val66Val and Met carrier. Box-and-whisker diagram in gray: the Val66Val group; in white: the Met carrier group. The %TRV of the Val66Val group was significantly higher than that of the Met carrier group in 2 of 47 regions. (***C***) Differences in GM volume variability between Female-Val66Val, Female-Met carrier, Male-Val66Val, and Male-Met carrier. The box-and-whisker diagrams represent Female-Val66Val, Female-Met carrier, Male-Val66Val, and Male-Met carrier groups from left to right, respectively. The %TRV of the Female-Val66Val group was significantly higher than that of the Male-Met carrier group in 3 of 47 regions.

The median %TRV of each brain region in women was 0.82–7.79% and the mean %TRV of the 47 regions was 2.24%, while the median %TRV of each brain region in men was 0.79–5.95% and the mean %TRV of the 47 regions was 1.88%. the Mann–Whitney test revealed that the %TRV in women was significantly higher than that in men in 5 of 47 regions (inferior frontal gyrus, opercular part: *P* = 0.001; middle occipital gyrus: *P* = 0.036; median cingulate and paracingulate gyri: *P* = 0.007; putamen: *P* = 0.047; pallidum: *P* = 0.014) (Fig. 3*A*).

### The Effect of the BDNF Genotype on GM Volume Variability

The median %TRV of each brain region in the Val66Val group was 0.91–6.61% and the mean %TRV of the 47 regions was 2.09%, while the median %TRV of each brain region in the Met carrier group was 0.90–7.71% and the mean %TRV of the 47 regions was 1.99%. The Mann–Whitney test revealed that the %TRV of the Val66Val group was significantly higher than that of the Met carrier group in 2 of 47 regions (inferior frontal gyrus, triangular part: *P* = 0.043; inferior frontal gyrus, orbital part: *P* = 0.025) (Fig. 3*B*).

### The Effect of Gender and BDNF Genotype on GM Volume Variability

The median %TRV of each brain region in the Female-Val66Val group was 0.77–5.59% and the mean %TRV of the 47 regions was 2.26%. The median %TRV of each brain region in the Female-Met carrier group was 0.87–8.45% and the mean %TRV of the 47 regions was 2.22%. The median %TRV of each brain region in the Male-Val66Val group was 0.73–7.26% and the mean %TRV of the 47 regions was 2.04%. The median %TRV of each brain region in the Male-Met carrier group was 0.65–8.20% and the mean %TRV of the 47 regions was 1.81%. The Kruskal–Wallis test revealed that the null hypothesis was rejected in 5 of 47 regions (inferior frontal gyrus, opercular part: *P* = 0.005; superior temporal gyrus: *P* = 0.039; putamen: *P* = 0.047; pallidum: *P* = 0.040; median cingulate and paracingulate gyri: *P* = 0.016). The Dann-Bonferroni test revealed that the %TRV of the Female-Val66Val group was significantly higher in 3 of 47 regions than the others (inferior frontal gyrus, opercular part: *P* = 0.037 (vs. Male-Met carrier group); superior temporal gyrus: *P* = 0.036 (vs. Male-Met carrier group); median cingulate and paracingulate gyri: *P* = 0.011 (vs. Male-Met carrier group) (Fig. 3*C*). Moreover, the %TRV of the Female-Met carrier group was significantly higher in the inferior frontal gyrus, opercular part than that of the Male-Met carrier group (*P* = 0.008).

We checked the effect size and power (1 − β) of the Kruskal–Wallis test for the brain regions that showed significant differences according to the results of this test. Results showed that two regions, the inferior frontal gyrus opercular part [effect size *f* = 0.570, power (1 − β) = 0.840] and superior temporal gyrus [effect size *f* = 0.685, power (1 − β) = 0.952] had good power, whereas, the median cingulate and paracingulate gyri had slightly low power [effect size *f* = 0.500, power (1 − β) = 0.722].

## Discussion

We examined the short-term (several months) variability of GM volumes in young healthy adults. Our results showed that the GM volume variability in 46 of 47 regions was small (ICC (1, 2) > 0.80). However, that in the thalamus was slightly high (ICC(1, 2) = 0694). In addition, we examined the effects of factors expected to contribute to the variability (gender and BDNF-genotype) on the GM volume variability. We could observe that the GM volume variability in women was higher than that in men, and in the Val66Val group than that in the Met carrier group. Moreover, the GM volume variability in the Female-Val66Val group was higher than in other groups. These results indicate that the short-term GM volume variability is small and stable, while it is affected by within-subject factors (Gender and BDNF-genotype). In particular, Female-Val66Val exhibited high GM volume variability.

### GM Volume Variability in all Subjects

The GM volume variability in 46 of 47 regions was small. Seiger et al. reported that the %TRV of GM volumes of healthy young adults for 3 months showed less than 3% in 42 of 46 regions (91%) (Seiger et al. 2015). Similarly, the %TRV showed less than 3% in 43 out of 47 regions (91%) in this study. However, the mean %TRV in this study (2.1% ± 1.3%) was slightly higher than that reported by Seiger et al. (1.6% ± 0.8%). This could be presumably due to the differences in sample size and imaging intervals. The sample size of the previous study comprised 10 participants, while that of this study 41. In addition, the imaging interval in the previous study was 81 ± 49 days, while in this study it was 115 ± 43 days. The mean %TRV value may have been higher due to the sample size and the imaging intervals were larger than those in the previous study. However, in all regions except the thalamus, the variability was small and the ICC was high in more than 90% of the brain regions, indicating that the short-term GM volume variability in young healthy adults are small and stable even when the sample size is increased. The results of the VBM analysis are strongly influenced by the magnetic field strength and imaging sequence of the MRI system used for T1-weighted brain imaging (Seiger et al. 2015;Okubo et al. 2016 ; Yan et al. 2020). In particular, setting the imaging conditions for T1-weighted brain imaging should be carefully executed as the structures located in the subcortical region tend to reduce the GM segmentation accuracy (Okubo et al. 2016).The GM volume variability in the basal ganglia was smaller using the magnetization-prepared two rapid acquisition gradient echo (MP2RAGE) sequence than using the MPRAGE sequence (Okubo et al. 2016; Yan et al. 2020), while we used the MPRAGE sequence in this study. The results of this study suggest that it would be necessary to consider the imaging conditions used for the VBM analysis depending on the target brain region.

### The Effect of Gender and the BDNF Genotype on GM Volume Variability

The GM volume variability in women was higher than in men, and in the Val66Val group than in the Met carrier group. Moreover, the GM volume variability in the Female-Val66Val group was higher than in the other groups. The GM volume in multiple brain regions changes with the menstrual cycle in women, which is thought be induced by changes in hormone levels associated with the changes in the menstrual cycle (Catenaccio et al. 2016; Meeker et al. 2020). Previous studies in mice have shown that elevated estrogen levels increase the dendritic density of hippocampal neurons, while elevated progesterone levels decrease it (Woolley and McEwen 1993; Murphy et al. 1998a; Murphy et al. 1998b). In addition, previous studies in humans reported that changes in GM volumes with menstrual cycle were related to changes of hormone levels (Lisofsky et al. 2015; Pletzer et al. 2018). Therefore, the high GM volume variability observed in women in this study might be caused by the changes in the hormone levels associated with the menstrual cycle. Recently, it has been suggested that this estrogenic effect might be caused by the direct effect of estrogen itself and the indirect effect mediated by BDNF (Begliuomini et al. 2007; Wu et al. 2013). Estrogen activates estrogen response elements (EREs) by binding to estrogen receptors in neuronal nuclei (Levin 2001). ERE is expressed in genes that regulate BDNF activity as estrogen promotes BDNF secretion by activating BDNF genes via the ERE (Sohrabji et al. 1995). Ovariectomy decreases estrogen levels and blood BDNF in Val66Val group mice, whereas it decreases estrogen levels but not blood BDNF levels in Val66Met group mice (Wu et al. 2015; McCarthny et al. 2018). In other words, estrogen-dependent BDNF secretion is higher in Val66Val than in Val66Met. Therefore, it could be suggested changes in estrogen levels during the menstrual cycle might have a stronger effect on the GM volume in the Val66Val group as the amount of the released estrogen-dependent BDNF is more relevant in the case of the Val66Val group. Therefore, the high GM volume variability could be observed in the Female-Val66Val group. However, it remains unclear whether changes in the menstrual cycle could contribute to the GM volume variability as the menstrual cycle of female participants was not investigated in this study. Furthermore, information about each subject’s life, such as their exercise habits, was not collected in this study. Further investigation would be required to examine the relationship between the changes in blood estrogen and BDNF concentration levels and those of GM volumes, and data on each subject’s life must also be collected.

The sample size for this study was 41, which is not the ideal sample size. However, the power (1 − β) of the Kruskal–Wallis test was good for the two brain regions that showed significant differences according to the Kruskal–Wallis. Therefore, the statistical results of the two regions in which significant differences were observed in this study can be trusted, although we cannot deny the possibility of Type II errors due to lack of detection in other brain regions. Thus, the results of this study could provide important insights if the data are interpreted with caution.

## Conclusion

We investigated the short-term variability of GM volumes in healthy young adults and the effect of Gender and BDNF genotype on the phenomenon. The short-term GM volume variability was small and stable, while it was affected by gender and BDNF-genotype. In particular, Female-Val66Val exhibited high GM volume variability.

## Notes

The authors would like to thank Enago (http://www.enago.jp) for the English language review. *Conflict of Interest:* The authors declare no competing interests.

## Data and code availability statement

The data that support the findings of this study are available from the corresponding author, [HW], upon reasonable request.

## References

[ref1] Ashburner J, Friston KJ. 2000. Voxel-based morphometry--the methods. Neuroimage. 11:805–821. 10860804 10.1006/nimg.2000.0582

[ref2] Begliuomini S, Casarosa E, Pluchino N, Lenzi E, Centofanti M, Freschi L, Pieri M, Genazzani AD, Luisi S, Genazzani AR. 2007. Influence of endogenous and exogenous sex hormones on plasma brain-derived neurotrophic factor. Hum Reprod. 22:995–1002. 17251358 10.1093/humrep/del479

[ref3] Catenaccio E, Mu W, Lipton ML. 2016. Estrogen- and progesterone-mediated structural neuroplasticity in women: evidence from neuroimaging. Brain Struct Funct. 221:3845–3867. 26897178 10.1007/s00429-016-1197-x PMC5679703

[ref4] Ceccarelli A, Rocca MA, Pagani E, Falini A, Comi G, Filippi M. 2009. Cognitive learning is associated with gray matter changes in healthy human individuals: a tensor-based morphometry study. Neuroimage. 48:585–589. 19615452 10.1016/j.neuroimage.2009.07.009

[ref5] Cirillo J, Hughes J, Ridding M, Thomas PQ, Semmler JG. 2012. Differential modulation of motor cortex excitability in BDNF met allele carriers following experimentally induced and use-dependent plasticity. Eur J Neurosci. 36:2640–2649. 22694150 10.1111/j.1460-9568.2012.08177.x

[ref6] Cohen J . 1992. Statistical power analysis. Curr Dir Psychol Sci. 1:98–101.

[ref7] Driemeyer J, Boyke J, Gaser C, Buchel C, May A. 2008. Changes in gray matter induced by learning--revisited. PLoS One. 3:e2669. 18648501 10.1371/journal.pone.0002669 PMC2447176

[ref8] Egan MF, Kojima M, Callicott JH, Goldberg TE, Kolachana BS, Bertolino A, Zaitsev E, Gold B, Goldman D, Dean M. 2003. The BDNF val66met polymorphism affects activity-dependent secretion of BDNF and human memory and hippocampal function. Cell. 112:257–269. 12553913 10.1016/s0092-8674(03)00035-7

[ref9] Gao X, Smith GM, Chen J. 2009. Impaired dendritic development and synaptic formation of postnatal-born dentate gyrus granular neurons in the absence of brain-derived neurotrophic factor signaling. Exp Neurol. 215:178–190. 19014937 10.1016/j.expneurol.2008.10.009

[ref10] Granert O, Peller M, Gaser C, Groppa S, Hallett M, Knutzen A, Deuschl G, Zeuner KE, Siebner HR. 2011. Manual activity shapes structure and function in contralateral human motor hand area. Neuroimage. 54:32–41. 20708692 10.1016/j.neuroimage.2010.08.013

[ref11] Hamzei F, Glauche V, Schwarzwald R, May A. 2012. Dynamic gray matter changes within cortex and striatum after short motor skill training are associated with their increased functional interaction. Neuroimage. 59:3364–3372. 22108643 10.1016/j.neuroimage.2011.10.089

[ref12] Hattemer K, Knake S, Reis J, Rochon J, Oertel WH, Rosenow F, Hamer HM. 2007. Excitability of the motor cortex during ovulatory and anovulatory cycles: a transcranial magnetic stimulation study. Clin Endocrinol (Oxf). 66:387–393. 17302873 10.1111/j.1365-2265.2007.02744.x

[ref13] Jing B, Liu B, Li H, Lei J, Wang Z, Yang Y, Sun PZ, Xue B, Liu H, Xu ZD. 2018. Within-subject test-retest reliability of the atlas-based cortical volume measurement in the rat brain: a voxel-based morphometry study. J Neurosci Methods. 307:46–52. 29960027 10.1016/j.jneumeth.2018.06.022 PMC6461491

[ref14] Kleim JA, Chan S, Pringle E, Schallert K, Procaccio V, Jimenez R, Cramer SC. 2006. BDNF val66met polymorphism is associated with modified experience-dependent plasticity in human motor cortex. Nat Neurosci. 9:735–737. 16680163 10.1038/nn1699

[ref15] Koo TK, Li MY. 2016. A guideline of selecting and reporting Intraclass correlation coefficients for reliability research. J Chiropr Med. 15:155–163. 27330520 10.1016/j.jcm.2016.02.012 PMC4913118

[ref16] Landi SM, Baguear F, Della-Maggiore V. 2011. One week of motor adaptation induces structural changes in primary motor cortex that predict long-term memory one year later. J Neurosci. 31:11808–11813. 21849541 10.1523/JNEUROSCI.2253-11.2011 PMC3180815

[ref17] Levin ER . 2001. Cell localization, physiology, and nongenomic actions of estrogen receptors. J Appl Physiol. 91:1860–1867. 11568173 10.1152/jappl.2001.91.4.1860

[ref18] Lisofsky N, Martensson J, Eckert A, Lindenberger U, Gallinat J, Kuhn S. 2015. Hippocampal volume and functional connectivity changes during the female menstrual cycle. Neuroimage. 118:154–162. 26057590 10.1016/j.neuroimage.2015.06.012

[ref19] Lovden M, Wenger E, Martensson J, Lindenberger U, Backman L. 2013. Structural brain plasticity in adult learning and development. Neurosci Biobehav Rev. 37:2296–2310. 23458777 10.1016/j.neubiorev.2013.02.014

[ref20] McCarthny CR, Du X, Wu YC, Hill RA. 2018. Investigating the interactive effects of sex steroid hormones and brain-derived neurotrophic factor during adolescence on hippocampal NMDA receptor expression. Int J Endocrinol. 2018:7231915. 29666640 10.1155/2018/7231915 PMC5831834

[ref21] McHughen SA, Rodriguez PF, Kleim JA, Kleim ED, Marchal Crespo L, Procaccio V, Cramer SC. 2010. BDNF val66met polymorphism influences motor system function in the human brain. Cereb Cortex. 20:1254–1262. 19745020 10.1093/cercor/bhp189 PMC2852510

[ref22] Meeker TJ, Veldhuijzen DS, Keaser ML, Gullapalli RP, Greenspan JD. 2020. Menstrual cycle variations in Gray matter volume, white matter volume and functional connectivity: critical impact on parietal lobe. Front Neurosci. 14:594588. 33414702 10.3389/fnins.2020.594588 PMC7783210

[ref23] Murphy DD, Cole NB, Greenberger V, Segal M. 1998a. Estradiol increases dendritic spine density by reducing GABA neurotransmission in hippocampal neurons. J Neurosci. 18:2550–2559. 9502814 10.1523/JNEUROSCI.18-07-02550.1998 PMC6793090

[ref24] Murphy DD, Cole NB, Segal M. 1998b. Brain-derived neurotrophic factor mediates estradiol-induced dendritic spine formation in hippocampal neurons. Proc Natl Acad Sci U S A. 95:11412–11417. 9736750 10.1073/pnas.95.19.11412 PMC21656

[ref25] Okubo G, Okada T, Yamamoto A, Kanagaki M, Fushimi Y, Okada T, Murata K, Togashi K. 2016. MP2RAGE for deep gray matter measurement of the brain: a comparative study with MPRAGE. J Magn Reson Imaging. 43:55–62. 26032895 10.1002/jmri.24960

[ref26] Onishi H, Otsuru N, Kojima S, Miyaguchi S, Saito K, Inukai Y, Yamashiro K, Sato D, Tamaki H, Shirozu H et al. 2018. Variability and reliability of paired-pulse depression and cortical oscillation induced by median nerve stimulation. Brain Topogr. 31:780–794. 29737438 10.1007/s10548-018-0648-5 PMC6097743

[ref27] Park H, Poo MM. 2013. Neurotrophin regulation of neural circuit development and function. Nat Rev Neurosci. 14:7–23. 23254191 10.1038/nrn3379

[ref28] Petersen N, Kilpatrick LA, Goharzad A, Cahill L. 2014. Oral contraceptive pill use and menstrual cycle phase are associated with altered resting state functional connectivity. Neuroimage. 90:24–32. 24365676 10.1016/j.neuroimage.2013.12.016 PMC4113343

[ref29] Pletzer B, Harris T, Hidalgo-Lopez E. 2018. Subcortical structural changes along the menstrual cycle: beyond the hippocampus. Sci Rep. 8:16042. 30375425 10.1038/s41598-018-34247-4 PMC6207699

[ref30] Poo M -m. 2001. Neurotrophins as synaptic modulators. Nat Rev Neurosci. 2:24–32. 11253356 10.1038/35049004

[ref31] Sasaki R, Otsuru N, Miyaguchi S, Kojima S, Watanabe H, Ohno K, Sakurai N, Kodama N, Sato D, Onishi H. 2021. Influence of brain-derived neurotrophic factor genotype on short-latency afferent inhibition and motor cortex metabolites. Brain Sci. 11:395. 10.3390/brainsci11030395 PMC8003639 33804682

[ref32] Schloemer N, Lenz M, Tegenthoff M, Dinse HR, Hoffken O. 2020. Parallel modulation of intracortical excitability of somatosensory and visual cortex by the gonadal hormones estradiol and progesterone. Sci Rep. 10:22237. 33335211 10.1038/s41598-020-79389-6 PMC7747729

[ref33] Seiger R, Hahn A, Hummer A, Kranz GS, Ganger S, Kublbock M, Kraus C, Sladky R, Kasper S, Windischberger C et al. 2015. Voxel-based morphometry at ultra-high fields. A comparison of 7T and 3T MRI data. Neuroimage. 113:207–216. 25791781 10.1016/j.neuroimage.2015.03.019 PMC5341769

[ref34] Smith MJ, Keel JC, Greenberg BD, Adams LF, Schmidt PJ, Rubinow DA, Wassermann EM. 1999. Menstrual cycle effects on cortical excitability. Neurology. 53:2069–2069. 10599783 10.1212/wnl.53.9.2069

[ref35] Sohrabji F, Miranda RC, Toran-Allerand CD. 1995. Identification of a putative estrogen response element in the gene encoding brain-derived neurotrophic factor. Proc Natl Acad Sci U S A. 92:11110–11114. 7479947 10.1073/pnas.92.24.11110 PMC40581

[ref36] Stein M, Federspiel A, Koenig T, Wirth M, Strik W, Wiest R, Brandeis D, Dierks T. 2012. Structural plasticity in the language system related to increased second language proficiency. Cortex. 48:458–465. 21106192 10.1016/j.cortex.2010.10.007

[ref37] Teutsch S, Herken W, Bingel U, Schoell E, May A. 2008. Changes in brain gray matter due to repetitive painful stimulation. Neuroimage. 42:845–849. 18582579 10.1016/j.neuroimage.2008.05.044

[ref38] Tzourio-Mazoyer N, Landeau B, Papathanassiou D, Crivello F, Etard O, Delcroix N, Mazoyer B, Joliot M. 2002. Automated anatomical labeling of activations in SPM using a macroscopic anatomical parcellation of the MNI MRI single-subject brain. Neuroimage. 15:273–289. 11771995 10.1006/nimg.2001.0978

[ref39] Woolley CS, McEwen BS. 1993. Roles of estradiol and progesterone in regulation of hippocampal dendritic spine density during the estrous cycle in the rat. J Comp Neurol. 336:293–306. 8245220 10.1002/cne.903360210

[ref40] Wu YC, Hill RA, Gogos A, van den Buuse M. 2013. Sex differences and the role of estrogen in animal models of schizophrenia: interaction with BDNF. Neuroscience. 239:67–83. 23085218 10.1016/j.neuroscience.2012.10.024

[ref41] Wu YW, Du X, van den Buuse M, Hill RA. 2015. Analyzing the influence of BDNF heterozygosity on spatial memory response to 17beta-estradiol. Transl Psychiatry. 5:e498. 25603414 10.1038/tp.2014.143 PMC4312832

[ref42] Yan S, Qian T, Marechal B, Kober T, Zhang X, Zhu J, Lei J, Li M, Jin Z. 2020. Test-retest variability of brain morphometry analysis: an investigation of sequence and coil effects. Ann Transl Med. 8:12. 32055603 10.21037/atm.2019.11.149 PMC6995743

